# Necdin, one of the important pathway proteins in the regulation of osteosarcoma progression by microRNA-200c

**DOI:** 10.1080/21655979.2022.2056693

**Published:** 2022-03-25

**Authors:** Jian Li, Zhuangzhuang Wu, Jiani Wang, Taiyong Wu, Zhen Shen, Long Zhang, Jia Lv, Junjun Bai, Yi Feng

**Affiliations:** a Second Clinical Medical College, Shanxi Medical University, Taiyuan, ShanXi, China; b Department of Orthopaedics, The Second Affiliated Hospital of Shanxi Medical University, Taiyuan, Shanxi, China; c First Clinical Medical College, Shanxi Medical University, Taiyuan, ShanXi, China; d Department of Orthopaedics, Xiamen University, Xiamen, Fujian, China

**Keywords:** Osteosarcoma, microRNA-200c, necdin, proliferation, migration, invasion

## Abstract

MicroRNA-200c (miR-200c) generally acts as a tumor suppressor in multiple cancer types and a promising therapeutic target in tumorigenesis. However, only a few studies have explained the role of miR-200c in the development of osteosarcoma (OS). In this study, we investigated the role of miR-200c in OS progression and identified the regulatory pathway protein NDN involved in inhibiting the occurrence and development of OS. Firstly, we found that miR-200c is downregulated in OS cells and tissues. As well, *in vitro* and *in vivo* experiments showed that upregulating miR-200c inhibits the proliferation, invasion, metastasis of Saos-2 cells, promotes the apoptosis of Saos-2 cells and suppresses tumor growth in mice, indicating miR-200c plays a major role in regulating the OS progression. Furthermore, bioinformatics analysis showed that an anti-tumor protein, necdin (NDN), might be a potential target by miR-200c. To verify this hypothesis, we measured the expression level of NDN in OS cells and tissues and found NDN is downregulated, suggesting NDN is functional in OS progression. Moreover, we found that the expression levels of NDN and miR-200c in *in vivo* and *in vitro* experiments were positively correlated. However, the results of dual-luciferase reporter gene experiment showed miR-200c does not directly act on the 3ʹ untranslated region (UTR) of NDN gene, indicating that NDN might be an important pathway protein which regulates OS progression in the presence of miR-200c. Therefore, miR-200c/NDN could be potential targets for developing effective treatment against OS.

## Graphical abstract

**Figure:**
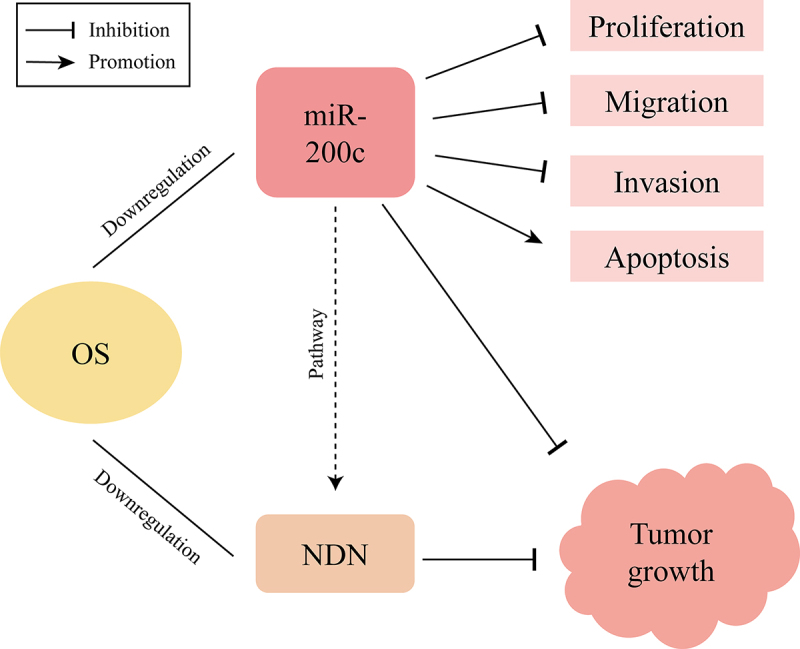

## Introduction

Osteosarcoma (OS), a malignant tumor, is mainly harmful to the health of children and adolescents and often occurs in the metaphysis of long bones (distal femur and proximal tibia) [1–4]. The high potential of its local recurrence and lung metastasis is the most important reason for its poor prognosis [5]. In recent years, certain comprehensive treatment methods, such as neoadjuvant chemotherapy and surgical therapy, have been developed, greatly improving the survival rate of patients with OS. However, these treatment methods still have several limitations in treating patients with lung metastasis, local recurrence and several systemic adverse reactions [2,6,7]. Consideration of the importance of targeted therapy methods which could decrease the incidence of lung metastasis and local recurrence and minimize certain systemic adverse reactions, significant efforts have been focused on finding new specific targeted molecules (nucleic acid fragments, protein products, etc.) on the surface or inside of the tumors. However, only a few therapeutic targets have been reported, limiting the efficiency of OS treatment.

MicroRNAs are a type of regulatory RNA sequences which can perform various biological functions by targeting relevant microRNAs [8]. MiR-200c, one of the microRNAs, acts as a tumor-suppressor and regulates various activities of tumors, such as proliferation, invasion, metastasis, and apoptosis [9]. Therefore, miR-200c is a potential therapeutic target for tumor treatments. Moreover, miR-200c regulates tumor progression by targeting several other proteins, such as metastasis associated lung denocarcinoma transcript 1 (MALAT1), hypoxia-inducible factor 1, alpha subunit (HIF-1a), homeodomain interacting protein kinase 1 (HIPK1)/β-catenin, checkpoint kinase 1 (CHK1) and fibroblast growth factor receptor substrate 2 (FRS2), which are also potential therapeutic targets for tumor treatments [10–13]. However, to the best of our knowledge, only threonine kinase 2 (AKT2) has been reported to be a target of miR-200c in OS [14]. Therefore, it is very instant to find another targeted pathway of miR-200c to accelerate the application of targeted therapy in OS treatment.

The gene encoding for NDN is located on the 15q11-13 chromosomal region which is associated with the development of Prader–Willi syndrome [15]. NDN plays important roles in regulating cell growth and gene transcription and the development of the nervous system. Among the members of the negative regulatory protein family, NDN is often associated with the progression of many tumors. Previous studies have reported that NDN can inhibit the development of colorectal cancer, ovarian cancer, head and neck squamous cell carcinoma, breast cancer, and bladder cancer, and its expression is downregulated in most cancers [16–20]. In terms of the molecular mechanism of NDN-associated carcinogenesis, Faveri, et al. found that NDN downregulation may induce NDN promoter hypermethylation and promote the activation of the Wnt signaling pathway to promote tumor cell proliferation [16,21]. However, the expression of NDN in OS has not been evaluated, and the role of miR-200c in NDN signaling and the progression of OS is still unclear.

In this study, we aimed to explore the role and molecular mechanism of miR-200c and NDN in OS development. We proposed a hypothesis that miR-200c overexpression suppresses the progression of OS by upregulating NDN. Our study provides a potential novel target for OS treatments.

## Materials and methods

### Human tissue samples

Human tissue samples were collected from 12 patients with OS who were diagnosed at the Department of Pathology of the Second Hospital of Shanxi Medical University. All the samples were collected during surgery and preserved in liquid nitrogen until further use.

### Cell culture

The human OS cell lines Saos-2 and U2-OS, and the human chondrocyte line C28/I2 were purchased from American Type Culture Collection (Manassas, VA, USA). The Saos-2 and U2-OS cell lines were cultured in Dulbecco’s modified eagle medium F-12 (DMEM/F-12), a complete medium supplemented with 10% fetal bovine serum and 1% penicillin [22]. The DMEM/F12 complete medium containing 10% fetal bovine serum was used to culture the C28/I2 cell lines. All the cells were cultured and maintained under the following conditions: 37°C, 5% CO_2_, and 95% humidity.

### Cell transfection

Saos-2 cells were cultured in a T25 culture flask in the logarithmic phase, inoculated in a 6-well plate at a concentration of 1 × 10^5^ cells per well, and cultured in a routine culture medium. Complete cell adhesion was achieved by incubating the plates overnight. The routine culture medium was replaced with HitrasnsG P infection solution diluted with the complete medium at a ratio of 1:25, with an average of 1 mL per well. Cells in the experimental and control groups were infected with lentivirus and a control virus, respectively, with the multiplicity of infection 10 (MOI 10). After 12 h of infection, the virus-infected cells were transferred to the conventional complete medium to continue the culture. The cells were divided into the control group and experimental group (miR-200c). After 72 h of infection, the cells were stained with a fluorescent dye. The effect of infection was observed by an inverted microscope. The infected cells was identified by Polymerase Chain Reaction (PCR) [23].

### Flow cytometric analysis

Flow cytometric was used to measure apoptosis. The Annexin V Phycoerythrin/7-amino-actinomycin D apoptosis detection kit (Sizhengbai, Beijing, China) was used for AnnexinV staining [24,25]. Briefly, 100 µL of cell suspension (control and miR-200c groups) was mixed with 5 µL of PE staining solution. The above mixtures were incubated in the dark environments for 5 min at room temperature. Then, the mixtures were added with 10 µL of 7-AAD staining solution and 400 µL of PBS buffer and immediately analyzed by flow cytometry (FACS Calibur System, BD, Franklin Lakes, NJ, USA).

### Cell proliferation assay

The transfected cells (control group and miR-200c group) were digested, and cell concentration was adjusted to 5000 cell/100 µL. Transfected cells (100 µL) were seeded onto 96-well plates, and then incubated for 30 min. After incubation, samples were analyzed by a real-time cell analysis (RTCA) workbench (ACEA Biosciences, USA) [26]. The number of cells was measured at 15 min intervals for 72 h.

### Migration assay

The cells in the control and miR-200c groups, which grew well after the transfection, were inoculated in a 6-well plate. Each group was provided with three multiple holes (1 × 10^5^ cells/well). When 90% confluence was achieved, we removed the 6-hole plate, drew three black lines horizontally on the outer surface of the bottom of each hole using a marker pen, scratched the cells using aseptic toothpicks, carefully washed off the cells scattered on the scratches with PBS, and added the complete culture medium to continue the culture. After 24 h, we removed the 6-hole plate to capture images for our records and to calculate the related area.

### Transwell assay

The transfected cells under good growth conditions and exhibiting logarithmic growth phase were washed with PBS after digestion and diluted to 30,000 cells/100 µL using serum-free DMEM/F12. A total of 100 µL of cell suspension was transferred into the upper Transwell chamber, and a complete DMEM/F12 culture medium was added to the lower Transwell chamber. The chamber was incubated for 24 h. The following procedure was performed after the 24-h incubation: cotton swab drying; treatment with 4% paraformaldehyde for 30 min; treatment with 1% crystal violet for 5 min; and PBS wash. The cells were observed using a microscope, and images were captured.

### PCR analysis

Total RNA was extracted from harvested cells or human tissues by a Trizol reagent extraction method (Thermo Fisher Scientific). To determine the expression of miR-200c, U6 expression was used as an endogenous reference. To determine the NDN expression, 18s was used as an internal control. The oligo primers were shown as follows: 5′-GGTAATACTCCCGGGTAAT-3’ (forward) and 5’-CAGTGCGTGTCGTGGAGT-3’ (reverse) for miR-200c, 5’-CGAGCTCATGTGGTACGT-3’ (forward) and 5’-CGATGACATCTTTCACCATGTC-3’ (reverse) for NDN. The data were normalized to those of the controls and analyzed by the 2^–ΔΔCt^ method [27].

### Western blot analysis

The radioimmunoprecipitation assay (RIPA) strong lysate (BOSTER, Wuhan, China) was used to extract the protein from cells and tissues. The 5× loading buffer (BOSTER, Wuhan, China) was used to dilute the extracted protein. According to the instructions provided for the SDS-PAGE gel preparation kit (BOSTER, Wuhan, China), 12% separation and 5% concentrated gels were used for electrophoresis, and then a semi-dry transfer system (conditions: 160 mA for 30 min) was used to transfer the protein onto a nitrocellulose membrane. Further, the protein was blocked, immunoreacted with anti-H2B (1:1000 dilution) and anti-NDN (1:100 dilution) antibodies, and exposed. The data were analyzed using the Image J software [28].

### Establishing an OS allograft mouse model

An OS allograft mouse model was established using nude mice and Saos-2 cells. In particular, male BALB/c nude mice (4–6-weeks-old) were purchased from Charles River Laboratory (Beijing, China) and maintained in a specific pathogen-free (SPF) conditions at a controlled temperature and humidity and alternating 12 h light and dark cycles for 7 days. All nude mice were subcutaneously inoculated with Saos-2 cells and was randomly divided into experimental (n = 6) and control (n = 6) groups. Animal handling and experimental procedures were in accordance with the Guide for the Care and Use of Laboratory Animals. miR-200c Agomir (Ruibo, Guangzhou, China) was injected subcutaneously into the tumors of nude mice in the experimental group at an injection dose of 2 nmol/mouse, and the control group received the same dose of normal PBS buffer. The dynamic growth of implanted tumors was monitored weekly, and the tumor growth curves were plotted accordingly (V = 1/6 × π × L× W^2^). At the end of the experiments (5 weeks), the mice were sacrificed, and their tumors were dissected. The tumor volumes and weights were measured. Tumor tissue samples were collected in 10% formalin, or RNA later solution or lysis buffer (stored at −80°C) for later use [29].

### Bioinformatics analysis

The potential targets of miR-200c and NDN were analyzed using specific programs in TargetScan (http://www.targetscan.org/vert_72/) and miRDB (http://www. mirdb.org/cgi-bin/search.cgi).The program shows the miR-200c binding site in the NDN 3ʹ-UTR region.

### Dual-luciferase reporter assay

The effects of miR-200c overexpression on the 3′-UTR of NDN was examined by a dual-luciferase reporter assay. Briefly, 293 T cells (1.0 × 10^4^ cells per well) were cultured in 96-well plates. A total of 5 µL of the OPTI-MEM medium (Gibco, Shanghai, China) was used to dilute miRNA mimics or non-target controls, 3′-UTR of NDN double reporter vector (NM_002487.3 complete 3UTR) or mutant vector, and 5 µL the OPTI-MEM medium (Gibco, Shanghai, China) was used to dilute 0.25 µL of the Lipo6000™ transfection reagent (Beyotime, Shanghai, China). The luciferase activity in individual wells was determined using the GLOMAX 20/20 luminometer (Promega) and the Dual-Luciferase Reporter Assay kit (Promega) according to the manufacturer’s instructions [30].

### Ethics statement

This study was reviewed by Shanxi Medical University and approved by the Ethics Committee of Shanxi Medical University (2017LL077), Taiyuan, Shanxi, China. Written informed consent was obtained from all the parents/legal guardians of all patients included in this study.

### Statistical analysis

All experiments were performed in triplicates, and data were analyzed using Prism 8 (GraphPad Software, La Jolla, CA, USA). Data are expressed as the mean ±standard error of the mean. Statistical evaluation for data analysis was performed using the Student’s *t*-test. P values <0.05 were considered statistically significant.

## Results

In this study, we aimed to investigate the function of miR-200c in the development of OS and the underlying molecular mechanisms. We hypothesized that miR-200c upregulation alleviated OS Progression by targeting NDN. We initially demonstrated that miR-200c is downregulated in OS cell lines and clinical OS tissue samples and found that miR-200c overexpression has great effects on the proliferation, migration, invasion, and apoptosis of OS cells *in vitro*. Also, through TargetScan database, we found that NDN was the potential target for miR-200c.Then With the up-regulation of miR-200c, the expression of NDN was also up-regulated *in vivo* and *in vitro* experiments of OS. But the results of dual-luciferase reporter gene experiment indicated that no specific site was involved in the direct interaction between miR-200c and NDN. The findings show that miR-200c and NDN have great potential for application in treatment of the OS.

### The expression level of miR-200c is down-regulated in human OS cell lines and tissues

**Figure 1.**
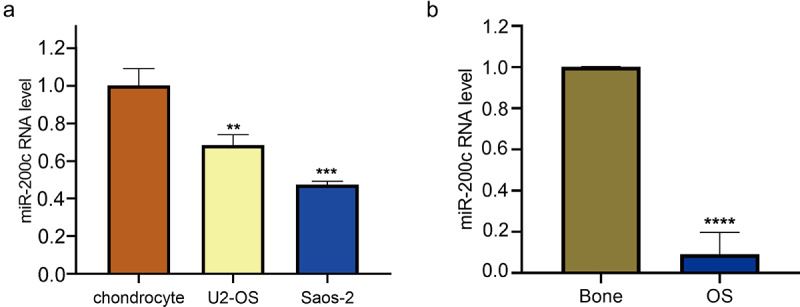
The relative expression level of miR-200c in human OS. (a) The relative expression level of miR-200c in chondrocyte, U2-OS and Saos-2 cells by qRT-PCR. (b) The relative expression level of miR-200c in 6 normal bone tissues and 12 OS tissues. Data are expressed as the mean ± SD or median and 75% percentile of each group from three separate experiments. **P < 0.01, ***P < 0.001, ****P < 0.0001.

To determine the role of miR-200c in OS, we firstly measured the expression of miR-200c in the OS cell lines (Saos-2 and U2-OS) and the normal human chondrocyte line C28/I2 by qRT-PCR. The results showed that the expression of miR-200c in OS cell lines was lower than that in chondrocyte line C28/I2 (P < 0.01, Figure 1(a)). Then, we evaluated the expression level of miR-200c in 12 OS tissue samples and 6 normal bone tissue samples. As shown in Figure 1(b), qRT-PCR results showed that the expression level of miR-200c in OS tissue samples was decreased when compared with the normal bone tissue samples (P < 0.0001). These results suggested that miR-200c expression was significantly down-regulated in the human OS cell lines and tissues.

### The effects of overexpression miR-200c on the proliferation, invasion, metastasis and apoptosis of Saos-2 cells

**Figure 2.**
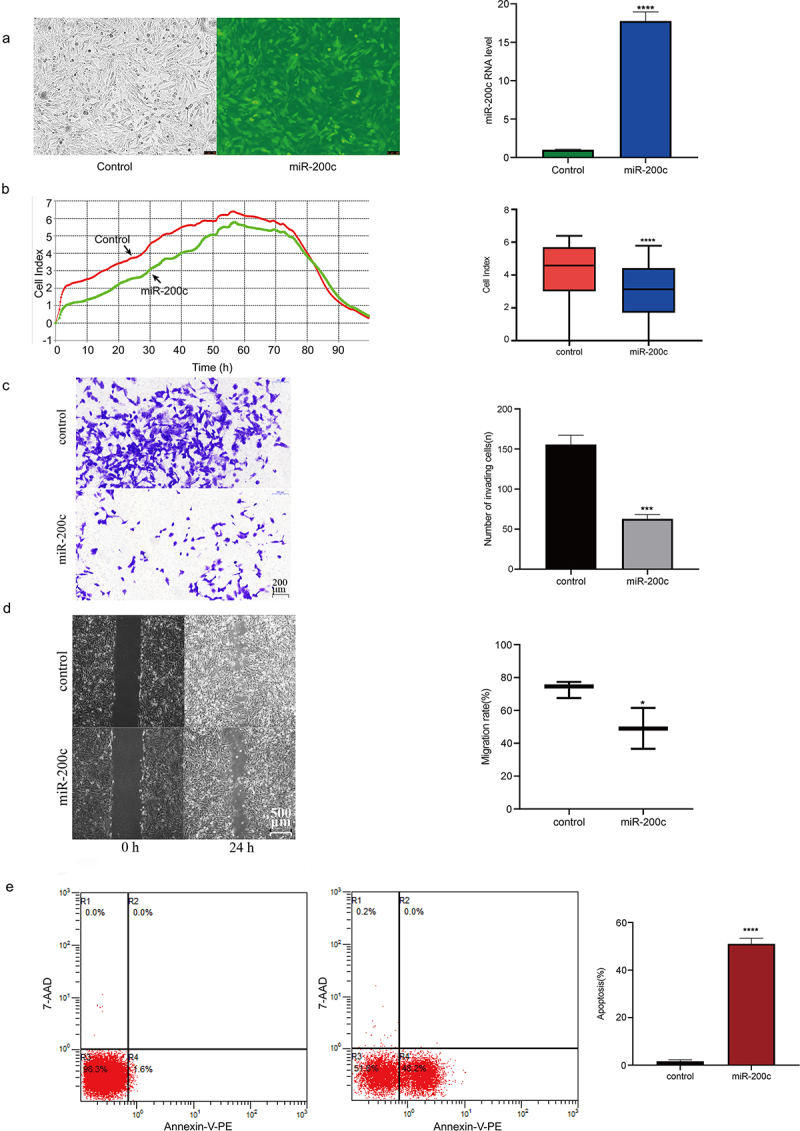
The effects of overexpression miR-200c on the proliferation, invasion, metastasis and apoptosis of Saos-2 cells in vitro. (a) Demonstration of the heterologous expression of miR-200c in Sao-2 cells by fluorescence microscope and qRT-PCR. (b) The proliferous results of control group and miR-200c overexpression group by RTCA xCELLigence system. (c) The invasive ability of control group and miR-200c overexpression group by Transwell assay. (d) The metastasizing ability of control group and miR-200c overexpression group by Migration assay. (e) Flow cytometry analysis of the percentage of apoptotic Saos-2 cells in control group and miR-200c overexpression group. Control group indicated that the Sao-2 cells without overexpression of miR-200c. Data are expressed as the mean ± SD or median and 75% percentile of each group from three separate experiments. *P < 0.05, ***P < 0.001, ****P < 0.0001.

To investigate the biological function of miR-200c during OS development, lentivirus was used to infect miR-200c into Saos-2 cells. The results of fluorescence microscope showed that more than 90% of the cells in the transfection group showed a green fluorescence (Figure 2(a)). Also, qPCR analysis showed that miR-200c has a higher expression level than that in control cells (P < 0.0001, Figure 2(a)). These results indicating the Saos-2 cells were successfully infected.

Next, we detected the effects of miR-200c overexpression on the cell proliferation, invasion, metastasis and apoptosis. Using the RTCA xCELLigence system, we found that the proliferative ability of Saos-2 cells in the miR-200c overexpression group was significantly weaker than that in the control group (P < 0.0001, Figure 2(b)), suggesting that overexpression of miR-200c *in vitro* inhibits the proliferation of OS cells. Similarly, the results of the transwell assay and migration assay showed that the ability of invasion (P < 0.001, Figure 2(c)) and metastasis (P < 0.05, Figure 2(d)) of OS cells in the miR-200c overexpression group was significantly attenuated when compared with the control group, suggesting that the overexpression of miR-200c *in vitro* also suppresses the invasion and migration of OS cells. Finally, we performed flow cytometric analysis to determine the effect of miR-200c overexpression on the apoptosis of OS cells. The results showed that the number of apoptotic cells in the miR-200c overexpression groups was higher than that in the control groups, especially in the early growth stage (48.4% vs.1.6%, P < 0.0001, Figure 2(e)), indicating that the overexpression of miR-200c promotes the cell apoptosis. All the above results demonstrated that miR-200c plays important role in inhibiting the proliferation, invasion, and metastasis of Saos-2 cells and promoting the apoptosis of Saos-2 cells *in vitro*.

### The effects of overexpression of miR-200c on tumor growth in mice

**Figure 3.**
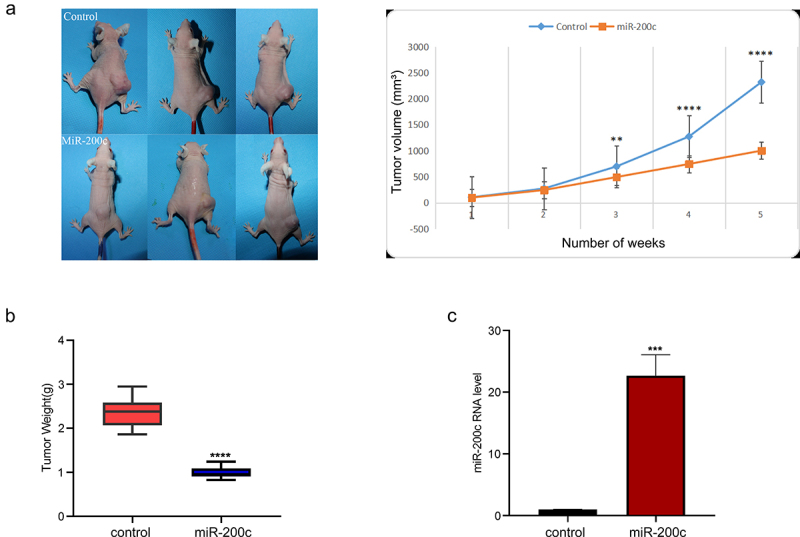
The effects of miR-200c on tumor growth in mice. (a) The dynamic growth (left) of the wildtype mice (control) and miR-200c overexpressed mice (miR-200c) and the volume (right) of the normal tumors (control) and the miR-200c implanted tumors (miR-200c) was monitored longitudinally. (b) At the end of the experiments, the weights of the formed tumor in wildtype mice (control) and miR-200c overexpressed mice (miR-200c) were measured. (c) qRT-PCR analysis of the relative RNA level of miR-200c in the normal tumors (control) and the miR-200c implanted tumors (miR-200c). Data are shown as the mean ± SEM of each group. ***P < 0.001, ****P < 0.0001.

To determine the effect of miR-200c overexpression on OS growth *in vivo*, an allogeneic tumor model of OS was established using nude mice and Saos-2 cells. The mice in the experimental group was injected with miR-200c agomir to increase the expression of miR-200c. The growth of the transplanted tumor was constantly monitored and recorded. The results showed that miR-200c overexpression significantly inhibited tumor growth (P < 0.001, Figure 3(a)) and reduced tumor weight (P < 0.001, Figure 3(b)). The expression of miR-200c in the transplanted tumor was determined by qPCR, and the results indicated that the expression of miR-200c in the experimental group was significantly increased compared with the control group (P < 0.001, Figure 3(c)). Our results indicated that miR-200c overexpression is responsible for inhibiting the growth of the implanted OS tumors *in vivo*.

### Demonstration of the interactions between miR-200c and NDN

**Figure 4.**
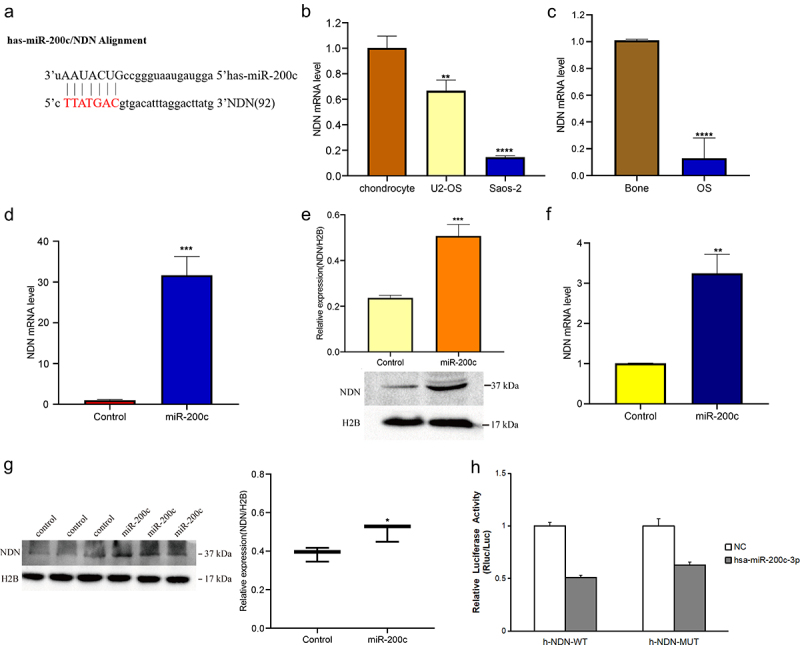
The expression of miR-200c on the expression of NDN in OS. (a) TargetScan and miRDB predicted that NDN is the potential target of miR-200c. (b) The expression level of NDN in chondrocyte, U2-OS and Saos-2 cells were determined by qRT-PCR. (c)The expression level of NDN in human OS (n = 12) and normal bone tissues (n = 6) were determined by qRT-PCR. (d) The relative transcription level of NDN in Sao-2 (control) and miR-200c overexpressed Saos-2 (miR-200c) cells were determined by qRT-PCR. (e) The relative expression of NDN in miR-200c overexpressed Saos-2 cells were detected by western blot. (g) The relative expression of NDN in miR-200c overexpressed mice (miR-200c) and the wild type mice (control) were determined by western blot. H2B is a reference gene. (h) The results of the dual-luciferase reporter gene experiment. Data are representative images or expressed as the mean ± SEM of each group from three separate experiments. *P < 0.05, **P < 0.01, ***P < 0.001, ****P < 0.0001.

Bioinformatics analysis showed that miR-200c and NDN are potential targets for preventing the development of OS (Figure 4(a)). To demonstrate the prediction, we firstly used qPCR to measure the expression level of NDN in the OS cells and tissues. As shown in Figure 4(b), the expression of NDN was significantly downregulated in the OS cell lines (P < 0.01). As well, the expression of NDN in the OS tissue samples was significantly decreased compared with the normal bone tissue samples (P < 0.0001, Figure 4(c)).

To further explore the potential interactions between miR-200c and NDN, we detected the NDN mRNA and protein expression level in the transfected cells in which the miR-200c was overexpressed and found that the expression of both NDN mRNA (P < 0.001, Figure 4(d)) and protein (P < 0.001, Figure 4(e)) was increased compared with the control cells. In addition, we also measured the mRNA and protein expression of NDN in nude mice in which miR-200c was overexpressed and found that the expression of both mRNA (P < 0.01, Figure 4(g)) and protein levels (P < 0.05, Figure 4(f)) in the tumors of nude mice was increased. To summarize, these results indicated that miR-200c overexpression promotes the expression of NDN in OS progression.

To confirm the interactions between miR-200c and NDN, we performed a dual-luciferase reporter gene experiment. The results showed that no specific site was involved in the direct interaction between miR-200c and NDN, suggesting that NDN may be an important pathway protein involved in the regulation of OS progression in the presence of miR-200c (Figure 4(h)).

## Discussion

Our findings showed that miR-200c inhibits the progression of OS by targeting NDN signaling. Our findings, such as the significantly downregulated expression of miR-200c in OS cells and tissues and inhibition of the occurrence and development of OS in vivo and in vitro due to the overexpression of miR-200c are consistent with the results of a previous study [14]. Furthermore, we report that miR-200c overexpression can significantly upregulate NDN expression in vivo and in vitro. Several studies have reported that NDN plays a tumor-suppressing role in various tumors [17–19]. Our results suggested that miR-200c may affect the occurrence and development of OS by regulating the expression of NDN, which has not been reported in a study of new therapeutic targets for OS [31]. Moreover, these results have also provided the required insight for identifying the pathway involving miR-200c that leads to inhibition of OS in subsequent studies. In the future, we aim to explore how NDN affects the development of OS and whether circular RNA is involved in the regulation of miR-200c expression.

Previous studies have reported that miR-200c targets the genes encoding MALAT1, HIF-1a, HIPK1/β-catenin, CHK1, FRS2, and PTEN to inhibit the occurrence and development of tumors [10–13]. Our bioinformatics analysis suggested that the 3’ UTR of NDN contains a miR-200c-binding motif, and our subsequent experiments proved that it is an important gene locus in the pathway of miR-200c for regulating the occurrence and development of OS. Our results extended the observations of previous studies and suggested that miR-200c may have more targets in the same type of tumor; thus, providing experimental ideas for further research. A study [32] based on breast cancer treatment showed that breast cancer patients with relatively high expression of miR-200c usually had a poor prognosis. In addition, miR-200c downregulation in our mouse model showed that it could effectively improve the outcome. The idea of using miR-200c as a blood biomarker for ovarian cancer has also been proposed [33]. These findings suggest that miR-200c is important for tumor diagnosis, and our study could be considered a reference for developing new diagnostic methods for OS.

NDN was firstly identified in patients with PWS and found to play an important role in the differentiation and survival of neurons [15]. NDN, a member of the MAGE(Melanoma-associated antigen) family [34], also encodes a protein that usually inhibits cell proliferation and acts as a transcriptional inhibitor. Moreover, several studies have reported low expression of NDN in colorectal cancer, ovarian cancer, head and neck squamous cell carcinoma, breast cancer, bladder cancer, and esophagus cancer [16–20,35]. It has also been reported to exhibit antitumor activity. However, the role of NDN in OS has not been reported, except for a genomic study that reported that NDN methylation plays an important role in OS development [36]. Some studies have suggested that NDN methylation may be an important cause of its anticancer effects [21,37]. NDN methylation was also observed in endometrial carcinoma by Liu [38]. Osaily [39] reported that miRNA can regulate the methylation of genes associated with breast cancer stem cells. Interestingly, a meta-analysis [40] indicated that the miR-200 family can cause NDN methylation, which is consistent with our results, and suggested that further exploration of the changes in NDN due to miR-200c is necessary.

## Conclusions

In the present study, we demonstrated that miR-200c is downregulated in OS cell lines and clinical OS tissue samples and found that miR-200c overexpression has great effects on the proliferation, migration, invasion, and apoptosis of OS cells *in vitro*. Also, we found that NDN was the potential target for miR-200c in OS by bioinformatics analysis, *in vivo* and *in vitro* experiments. The results show miR-200c/NDN could be potential targets for developing effective treatment methods against OS.
